# Laparoscopic Conversion of Single-Anastomosis Duodenal Switch (SADI-S) to Roux-en-Y Gastric Bypass With Concurrent Paraesophageal Hernia Repair for Refractory Biliary Reflux and Paraesophageal Hernia

**DOI:** 10.7759/cureus.36205

**Published:** 2023-03-15

**Authors:** Zachary M Wargel, Timothy W Ritchie, Emanuel Shapera, Andrew A Wheeler

**Affiliations:** 1 Bariatric Surgery, University of Missouri School of Medicine, Columbia, USA; 2 Surgery, Sharp Grossmont Hospital, La Mesa, USA

**Keywords:** complications, revisional surgery, paraesophageal hernia, biliary reflux, sadi-s

## Abstract

Single-anastomosis duodenal switch (SADI-S) is effective for weight loss with low reported rates of complications. Bile reflux into the stomach or esophagus is an uncommonly reported complication but can lead to significant symptoms for patients suffering from this complication. Concurrent paraesophageal hernia can exacerbate the symptoms of biliary reflux gastritis. We present a case report describing the management of biliary reflux gastritis with concurrent paraesophageal hernia, our decision-making process, and technical pearls and possible pitfalls.

## Introduction

Single-anastomosis duodenal switch (SADI-S) is an effective therapy for treating morbid obesity with favorable perioperative outcomes [1-4] and involves performing a sleeve gastrectomy (SG) with the addition of a loop anastomosis rather than second lower anastomosis as is required in a standard biliopancreatic diversion with duodenal switch. However, bile reflux, a significant risk factor for esophageal adenocarcinoma in non-bariatric surgery [5], has been shown to be more common in SADI-S than with standard duodenal switch due to the loop configuration of the duodenoileostomy vs. more distal anastomosis [6]. Here we present a case of bile reflux and concurrent paraesophageal hernia following SADI-S that was treated by conversion to a Roux-en-Y gastric bypass (RYGB) with paraesophageal hernia repair. We hope to raise awareness of this important complication of SADI-S and to improve the outcomes of patients by providing a viable treatment option.

This article was presented as a video abstract at the 2022 annual meeting of the American Society for Metabolic and Bariatric Surgery (ASMBS) in Dallas, Texas, on June 7, 2022.

## Case presentation

A 58-year-old male presented to the bariatric clinic with dysphagia and post-prandial epigastric pain. Two years prior, he had undergone SADI-S at an outside hospital for morbid obesity and successfully lost 122 lbs to a weight of 166 lbs and a body mass index of 26. Abdominal ultrasound revealed a distended gallbladder, and hepatobiliary scintigraphy demonstrated biliary dyskinesia. He underwent an uneventful laparoscopic cholecystectomy with intraoperative upper endoscopy and concurrent internal hernia repair for an incidental hernia identified at Petersen's space. The upper endoscopy demonstrated the absence of a pylorus, a 5 cm long hiatal hernia, and significant biliary reflux gastritis. Post-cholecystectomy, his symptoms continued, and it was felt his dysphagia was likely related to the paraesophageal hernia and the bile reflux was secondary to complications from the SADI-S, possibly exacerbated by what appeared to be an absence or dysfunction of a pylorus. It was thought to have been divided during duodenal transection at the index SADI-S operation.

Given these findings, the patient was offered laparoscopic conversion of SADI-S to an RYGB and concurrent hiatal hernia repair to treat symptoms of bile reflux and dysphagia. The paraesophageal hernia was repaired after the stomach was reduced into the abdomen, so 3 cm of the intraabdominal esophagus was present. The hiatus was repaired with 2-0 braided polyester sutures placed in an interrupted fashion. The patient's intestinal anatomy was then inspected, and the small intestine was measured to ensure adequate lengths of intestinal components of the gastric bypass were achieved. The efferent limb was 275 cm from the duodenoileostomy to the terminal ileum, and the proximal afferent limb from the ligament of Treitz to the anastomosis was 435 cm long. The duodenoileostomy was then transected both proximally at the level of the future gastric pouch and then proximal to the anastomosis on the afferent limb and distal to the anastomosis on the efferent limb. The two ends of the small bowel were reanastomosed in a laparoscopic hand-sewn manner. The gastric bypass was then completed with the creation of a 20 cm biliopancreatic limb and a 75 cm alimentary limb. A linear stapled gastrojejunostomy was then created with an appropriate-length gastric pouch. The procedure was uneventful with minimal blood loss and no intraoperative or postoperative complications. The video of the procedure can be seen in Video 1.

**Video 1:** Conversion SADI-S to RYGB Technique of conversion of SADI-S to RYGB for bile reflux and paraesophageal hernia repair SADI-S - single-anastomosis duodenal switch, RYGB - Roux-en-Y gastric bypass

Postoperatively, the patient had an uneventful recovery in the hospital and was discharged on postoperative day three. After discharge, the patient did well with complete resolution of his bile reflux and associated symptoms and dysphagia. At the three-month follow-up visit, which was his last follow-up, he continued with a resolution of preoperative symptoms. At the final follow-up, his weight was stable compared to his preoperative visit.

## Discussion

SADI-S was first described in 2007 and is an effective alternative to other bariatric operations due to outstanding weight loss and favorable complication rates [1,3,4,7]. Prior studies have shown SADI-S to result in shorter hospital stays and fewer long-term complications when compared to biliopancreatic diversion with duodenal switch (BPD-DS) [3,4]. However, in addition to the usual complications following bariatric surgery, such as an anastomotic leak, SADI-S has a known risk of postoperative bile reflux due to the loop configuration of the duodenoileostomy allowing bile to continue to come in contact with the anastomosis and the potential for reflux into the stomach [3,4,8].

When severe bile reflux develops, revisional surgery may be indicated both to decrease symptoms as well as decrease the possibility of biliary reflux leading to esophageal adenocarcinoma. However, esophageal cancer as a result of biliary reflux after SADI-S has not been reported. In a large cohort comparison of SADI-S to BPD-DS, 3/181 SADI-S patients developed significant gastroesophageal reflux disease due to bile reflux [4]. These patients underwent conversion to BPD-DS with no outcomes reported. BPD-DS, while proven to be an effective weight loss procedure, carries a higher risk of nutritional deficiencies [9,10]. Our patient's conversion to RYGB with a resolution of symptoms supports this as a possible treatment modality for post-SADI-S bile reflux. Although there are concerns for weight regain due to a shorter biliopancreatic limb, one study that utilized a 30cm biliopancreatic (BP) limb and a 150 cm Roux limb during gastric bypass suggested similar weight loss to SADI-S patients at 18 months [1]. However, in patients with RYGB, variation in BP limb length affects weight loss at 36 months [11], whereas Roux limb length variation has not been shown to affect weight loss at one year [12]. Although our patient was lost to follow-up after three months, at that time, he had not regained any weight. One contributing factor for conversion to a gastric bypass was the patient required concurrent paraesophageal hernia repair to treat his dysphagia. Hiatal hernia occurrence after sleeve gastrectomy, as well as recurrent hiatal hernia after sleeve gastrectomy, has been shown to have increased complications compared to those patients without recurrent hernia [13,14]. Although hiatal hernia repair has been described after sleeve gastrectomy and BPD-DS [15], symptomatic recurrences may be higher than repair in a patient who previously underwent a gastric bypass [16]. Furthermore, the conversion of a sleeve to bypass with concurrent hiatal hernia repair has been shown to be an effective treatment for refractory reflux disease after sleeve gastrectomy, particularly when patients have simultaneous hiatal hernia [17]. This may be because when the sleeve migrates into the mediastinum, any angulation or twisting of the stomach will likely lead to partial obstruction, whereas the short gastric pouch with a gastric bypass that migrates into the mediastinum in a recurrent hiatal hernia is less likely to become obstructed. Although not specifically evaluating recurrent hiatal hernias, one study showed concurrent hiatal hernia repair after sleeve versus no hiatal hernia repair showed patients undergoing hiatal hernia repair had increased rates of future abdominal surgery or postoperative endoscopies. Matched bypass patients with hiatal hernia repair versus no hiatal hernia repair showed only increased use of postoperative endoscopy [18]. 

Studies indicate that bile reflux may be of significant concern for patients after SADI-S. In a study of 1328 patients, only two cases (0.1%) of class II clinically significant bile reflux (based on the Clavien-Dindo grading system) were found after BPD-DS, whereas rates of biliary reflux after single anastomosis duodenoileostomy have been shown to occur in 7.5% of patients and represent the main indication for revisional surgery after SADI-S [6]. The true incidence of long-term complications after SADI-S have yet to be determined, as longer-term data is necessary. However, when complications do develop after SADI-S, surgeons caring for these patients need to have multiple revisional surgical options available as the different presentations create a need for multiple options.

Technical pearls

During revisional surgery involving the conversion of SADI-S to RYGB, a few important points should be considered. First, ensuring bowel lengths are appropriate will avoid too distal of a bypass which could place the patient at risk for pathologic intestinal malabsorption. Thus when converting a SADI-S to an RYGB, because of the short efferent limb, simply resecting the duodenoileostomy with subsequent Roux-en-Y reconstruction and creation of a small gastric pouch maintaining the afferent and efferent limbs at the same length may lead to significant malabsorption and nutritional deficiencies. This is secondary to two main causes. First, with increased restriction secondary to a small gastric pouch, adequate consumption of protein can be difficult in a patient with a very distal gastrointestinal anastomosis. Second, simply creating an anastomosis between the efferent limb and newly constructed gastric pouch and connecting the afferent limb more distally on the efferent limb for the creation of the enteroenterostomy will result in a 225 cm or less common channel if the standard 300 cm common channel was constructed during the index SADI-S. By anastomosing the efferent and afferent limbs at the point of division of the duodenoileostomy and then constructed lengths for the alimentary and biliopancreatic limbs, as for a standard gastric bypass, significant malabsorption can be avoided. Secondly, it should be noted that because the lengths of the bypassed intestines are less than in a SADI-S, weight regain to some extent is possible and the patient should be counseled to that extent. Patients should definitely understand that although they may be under the impression they can lose more weight after a gastric bypass based on described experiences of others, the gastric bypass has often been shown to be inferior in regards to weight loss compared to the SADI-S [19].

## Conclusions

SADI-S may result in an increased incidence of bile reflux, as well as other complications after the procedure. While there have been reports of SADI-S conversion to standard duodenal switch to mitigate the risk of cancer, this is the first case report to our knowledge demonstrating the efficacy of conversion to RYGB. In this case, additional presenting symptoms made RYGB anatomy a more viable option, but this highlights that revisional bariatric surgeons that care for complex patients need to understand the evaluation for complications after SADI-S and possible options to treat these issues when they arise.
